# Recognition of a Phase-Sensitivity OTDR Sensing System Based on Morphologic Feature Extraction

**DOI:** 10.3390/s150715179

**Published:** 2015-06-29

**Authors:** Qian Sun, Hao Feng, Xueying Yan, Zhoumo Zeng

**Affiliations:** Tianjin University, State Key Laboratory of Precision Measurement Technology & Instruments, 92 Weijin Road, Nankai District, Tianjin 300072, China; E-Mails: sunqiansohu@126.com (Q.S.); fightingyxy@126.com (X.Y.); zhmzeng@tju.edu.cn (Z.Z.)

**Keywords:** Φ-OTDR, morphology, feature extraction, intrusion event recognition

## Abstract

This paper proposes a novel feature extraction method for intrusion event recognition within a phase-sensitive optical time-domain reflectometer (Φ-OTDR) sensing system. Feature extraction of time domain signals in these systems is time-consuming and may lead to inaccuracies due to noise disturbances. The recognition accuracy and speed of current systems cannot meet the requirements of Φ-OTDR online vibration monitoring systems. In the method proposed in this paper, the time-space domain signal is used for feature extraction instead of the time domain signal. Feature vectors are obtained from morphologic features of time-space domain signals. A scatter matrix is calculated for the feature selection. Experiments show that the feature extraction method proposed in this paper can greatly improve recognition accuracies, with a lower computation time than traditional methods, *i.e.*, a recognition accuracy of 97.8% can be achieved with a recognition time of below 1 s, making it is very suitable for Φ-OTDR system online vibration monitoring.

## 1. Introduction

Optical fiber vibration sensing systems based on phase-sensitive optical time-domain reflectometer (Φ-OTDR) devices can detect and locate vibration signals by measuring the backscatter light scattered across the entire optical spectrum. A Φ-OTDR uses a single mode fiber for optical transmission and sensing, and can be used for real-time monitoring and accurate positioning across long distances [1,2]. It has been used for monitoring the health of engineering structures, optical fiber perimeter protection and gas or oil pipeline safety pre-warning systems [3,4,5,6]. Accurate recognition of different vibration signals is crucial in a Φ-OTDR pre-warning system. False positives will be an inefficient use of resources and more seriously, delays in processing time may threaten people’s lives and property, therefore, there is a lot of attention is being given in Φ-OTDR research to techniques to accurately recognize the event type, ensure warnings are given in sufficient time and reduce the false positive rate.

**Figure 1:**
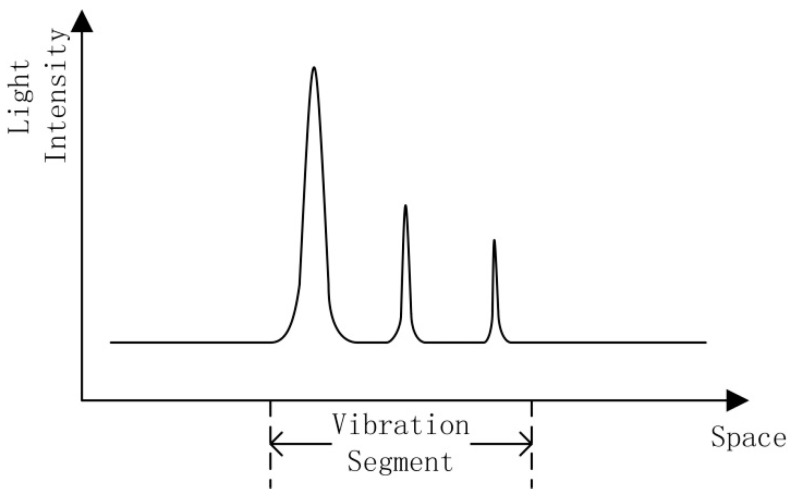
**Figure** **1.** Scattering curve of intrusion signal.

Due to the nonlinear, dynamic nature of the signal acquired by a Φ-OTDR vibration sensing system, a location scheme based on the wavelet packet transform (WPT) is proposed to reduce the number of false alarms [7]. Previous studies have used the short-time Fourier transform (STFT) and the continuous wavelet transform (CWT) for recognition of Φ-OTDR systems [8]. These traditional methods focus on finding and locating the intrusion events. If the features of the time domain signal can be extracted after the event has been located, different event types can be classified based on these signal features. However, this method is time consuming, due to the requirement to firstly pinpoint the location in the recognition process. If multiple events occur simultaneously, the recognition time will increase significantly. The intrusion signal of an Φ-OTDR system is not a single point in the space domain; it occurs across a range, since the attenuation will continue for a period along the optic fiber. Within the attenuation range, each scattered light signal contains the vibration response, but since the initial phase of each interference signal is different, the amplitudes of the vibration responses are different within the attenuation range, as shown in Figure 1. The peaks are due to backscattering when an intrusion event occurs. However, the maximum point may not be the location of the intrusion. If the event location is identified as the point with the maximum light intensity, then an error in location will occur.

Once there is an error in locating the event, the time domain signal of that location will not be the real signal corresponding to the intrusion event. Therefore the features of the event cannot be extracted correctly, which will lead to a recognition error. This conventional method is inadequate for a Φ-OTDR system and thus a highly efficient and precise method is necessary for recognition of intrusion events. In this paper, after a brief presentation of the Φ-OTDR signal characteristics in Section 2, a method based on time-space domain signals is proposed. The algorithm is evaluated in this paper using the criteria of recognition accuracy and speed. The recognition accuracy is the percentage of correct recognition events of all events. Recognition speed is the time calculated from feature extraction to recognition. The method proposed in this paper improves both the speed and accuracy of the recognition method, and is therefore a very suitable method to recognize different events within a Φ-OTDR sensing system. In Section 3, a feature extraction method based on morphology is proposed. The classifier design and performance evaluation are demonstrated in Section 4, and the final section provides the conclusions.

## 2. Φ-OTDR Signal Characteristics

**Figure 2:**
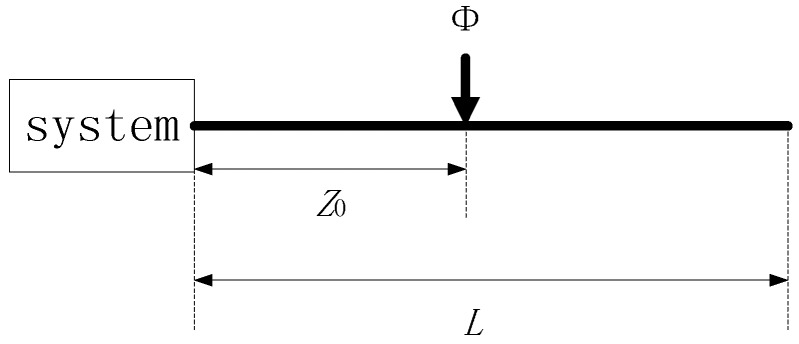
Simplified construction of a Φ-OTDR system.

The Φ-OTDR system has unique signal characteristics compared with other optic fiber sensing systems. Rayleigh Scattering (RS) light traveling within a fiber is phase modulated by vibrations that are applied to the fiber to acquire a RS curve for the pulse duration. The time-space domain signal is acquired by clustering all RS curves. It can reflect the characteristics in the space and time domain simultaneously. The propagation distance of vibrations in the space domain reflects the energy of the intrusion signal and the signal characteristics in the time domain reflect the duration of the intrusion event. A simplified construction of an Φ-OTDR system is shown in Figure 2. It can be assumed that that *ε*(*t*) is the optical pulse and *L* is the length of optical fiber. The vibration occurs at position *z*_0_ and the optical phase is therefore modulated by the vibration, with variation Φ.

The Rayleigh scattering field intensity can be expressed as follows:

(1) $$E(\phi ,s,n)=E_{1}(s,n)+E_{2}(s,n)\exp(2i\phi )$$

(2) $$E_{1}=\int_{0}^{z_{0}}\varepsilon (s-\frac{2z}{c})r(z)\exp(-2i\beta z)dz$$

(3) $$E_{2}=\int_{z_{0}}^{L}\varepsilon (s-\frac{2z}{c})r(z)\exp(-2i\beta z)dz$$

where *n* is the number of optical pulses. If the optical pulse period is T, *s* ∈ (*nT*, *nL*/*c*+*nT*). s can also be regarded as the abscissa of any point in the optical fiber. c is the velocity of light, β is the transmission constant, 2*z*/*c* is the optical pulse delay and 2β*z* is the optical phase delay. *E*_1_ and *E*_2_ are the Rayleigh scattering field intensities before and after the intrusion event, respectively.

The interference intensity can be expressed as:

(4) $$I\left(\phi ,s,n\right)={\left|E\left(\phi ,s,n\right)\right|}^{2}=I_{1}\left(s,n\right)+I_{2}\left(s,n\right)+2{\left[I_{1}\left(s,n\right)I_{2}\left(s,n\right)\right]}^{1/2}\cos\left(2\phi +\phi_{0}\right)$$

(5) $$\begin{array}{l} \phi_{0}=\arg\left(E_{2}\right)-\arg\left(E_{1}\right)\\ I_{i}={\left|E_{i}\right|}^{2},i=12. \end{array}$$

where *ϕ*_0_ is the initial phase of the Φ-OTDR system. The value of *ϕ* in Equation (4) not only changes in the time domain but also in the spatial domain. The attenuation of *ϕ* in the spatial domain can be expressed as:

(6) $$\phi (s,d)=\left\{ \begin{array}{ll} \Delta \delta e^{-\alpha (s_{0}-s)} & s<s_{0}\\ \Delta \delta e^{-\alpha (s-(s_{0}+d))} & s>(s_{0}+d)\\ \Delta \delta & s_{0}\leq s\leq (s_{0}+d) \end{array}\right.$$

**Figure 3:**
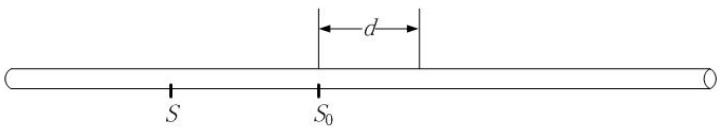
Parameters of Equation (6).

In Equation (6), α is the phase attenuation coefficient, *s*_0_ is the position of the intrusion event and *d* is the range of the intrusion force. Δδ is the phase variation in the time domain. The schematic diagram is shown in Figure 3.

The length and refractive index of the optical fiber will be modified when there is an intrusion force on the optical fiber. Δδ can be calculated as follows:

(7) $$\Delta \delta =\beta \Delta L+L\Delta \beta =\beta L\frac{\Delta L}{L}+L\frac{\partial \beta }{\partial n}\Delta n$$

where *β* is the propagation constant of the optical wave, *L* is the length of the optical fiber and *n* is the refractive index. The *βL*(Δ*L*/*L*) term is the effect due to strain, which can be regarded as being due to the phase variation along with the optical length variation. The L(∂β/∂n)Δn term is the photoelastic effect, due to the phase variation along with the refractive index variation. Δ*L* and Δ*n* are determined by the characteristics of the intrusion force and edatope, which are calculated in detail in the literature [9].

A Φ-OTDR pipeline safety pre-warning system was used as the case study in this paper. Common events which threaten pipeline safety are a vehicle passing over the pipeline, soil digging above the pipeline, and walking over the pipeline. Experiments were done using the Dagang-Zaozhuang product oil pipeline. The sensing cable used is a GYTA six-core single mode communication optical fiber, which was buried 30 cm above the pipeline and 1.5 m underground. Twenty km of optical fiber was used in the experiment. The laser pulse repetition frequency is 500 Hz and the sampling frequency is 50 MHz. Two seconds of data are used to constitute an image. For this particular application, the recognition accuracy must be as high as possible.

**Figure 4:**
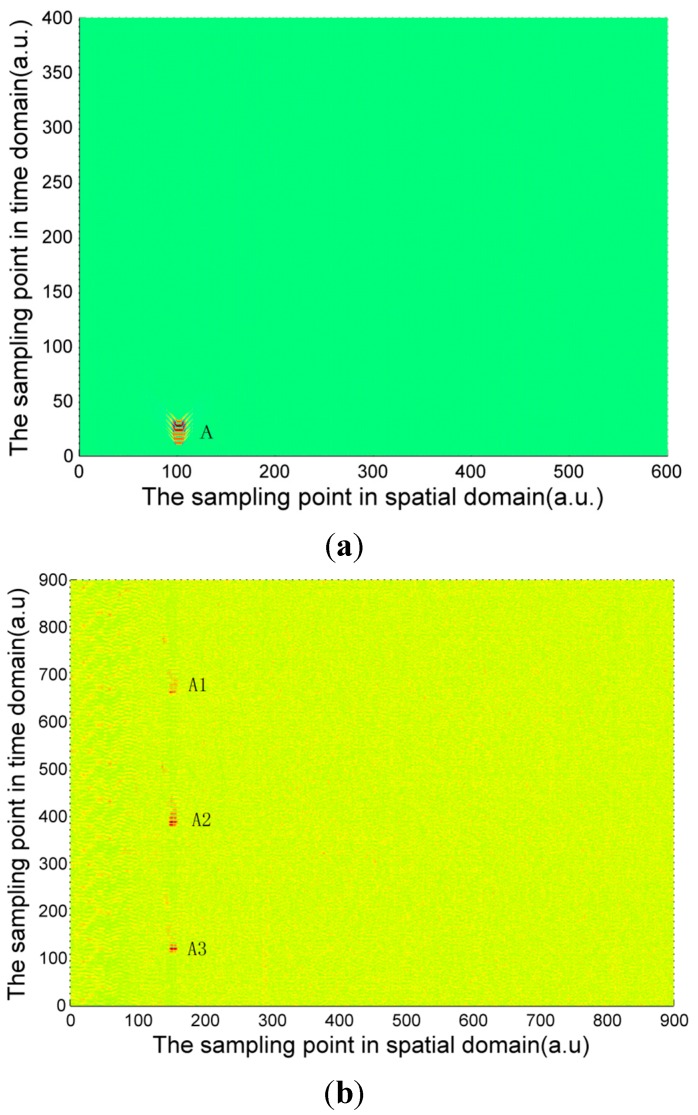
(**a**) Calculated walking signal; (**b**) Experimentally measured walking signal.

**Figure 5:**
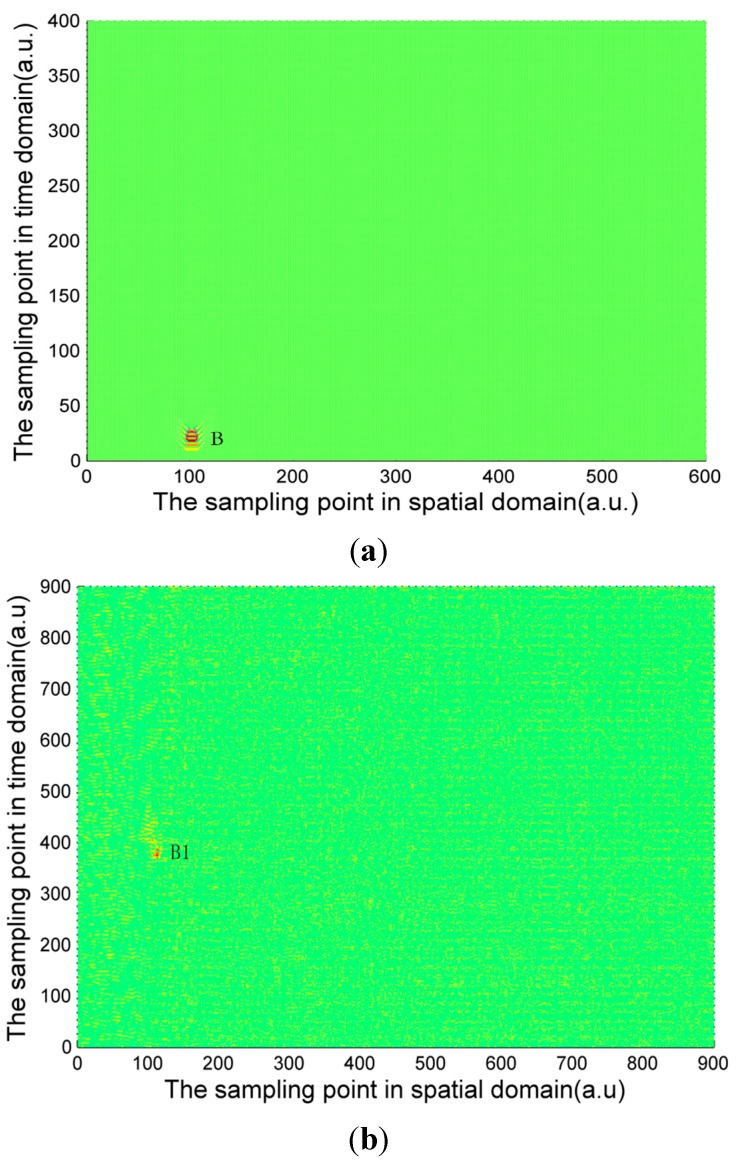
(**a**) Calculated digging signal (**b**) Experimentally measured digging signal.

**Figure 6:**
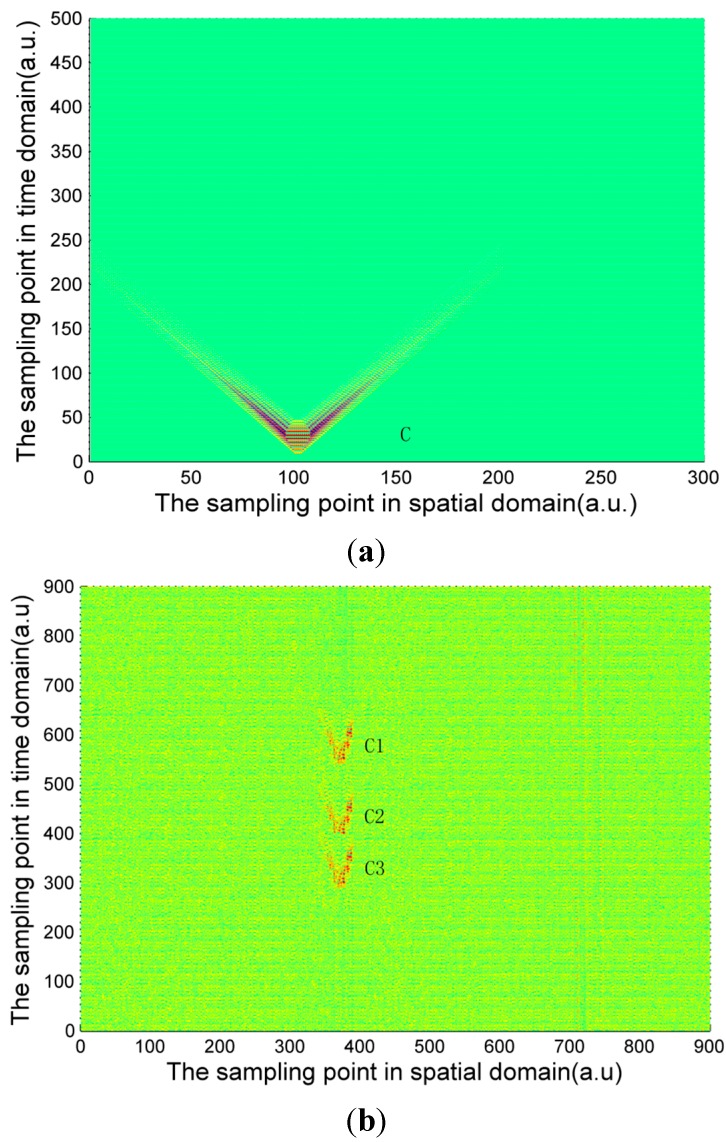
(**a**) Calculated signal of vehicle passing; (**b**) Experimentally measured signal of vehicle passing.

The signal calculated by Equation (4) and the experimentally measured signals of three pipeline safety events are shown in Figure 4, Figure 5 and Figure 6. Due to the long distance of the Φ-OTDR system monitoring, the vibration range is relatively small. An intrusion event is usually a fleck in the time-space domain image. The intrusion event occurs continuously in practice, so there are not only one event region in general in time-space signal image. Usually, walking frequency is three steps within 2 s, the interval between the wheels of the vehicle pressing the deceleration strip is about 0.25 s, and the interval due to digging is much larger than 2 s in the experiment. There are three event regions A1, A2, A3 in the experimental walking event, A2, A3 are repetition of A1. There are only one event region B1 in the experimental digging event, and three event regions C1, C2, C3 in the vehicle passing event. Therefore events are calculated only once for simulation signal, marked as region A, region B and region C in Figure 4, Figure 5 and Figure 6. In Figure 4, Figure 5 and Figure 6, the graphs in (a) are the simulation signals calculated by Equation (4) and the graphs in (b) are the experimentally measured signals. In order to illustrate the problem more clearly, the simulation signals are appropriately amplified. In the simulation process, for calculation convenience, many environmental factors were ignored. Soil is idealized as elastic half-space [10], and intrusion events are idealized as single frequency signals. Although there are some differences between the simulation and experimental signal images, they are basically the same.

The characteristic differences of the three types of events are obvious both in the simulation and experimental signals images. When a vehicle passes over the optical fiber, a vibration signal is produced by the wheels pressing on the deceleration strip on the ground. The duration of this event is very short but the energy is large, so the force can be considered ideally as an instantaneous force. Since the propagation distance is relatively high, a “V”-like shape will be formed. The forces due to walking and digging change relatively slowly with time and energy; the propagation distance is short. The energy and force variation of walking and digging are both different, so the size and the shape of the regions in the image are different. Based on the different image characteristics of each of the three different events, image processing technology can be used for image segmentation, and then a feature extraction method based on morphology proposed in this paper is used for event classification.

## 3. Feature Extraction Based on Morphology

### 3.1. Preprocessing

**Figure 7:**
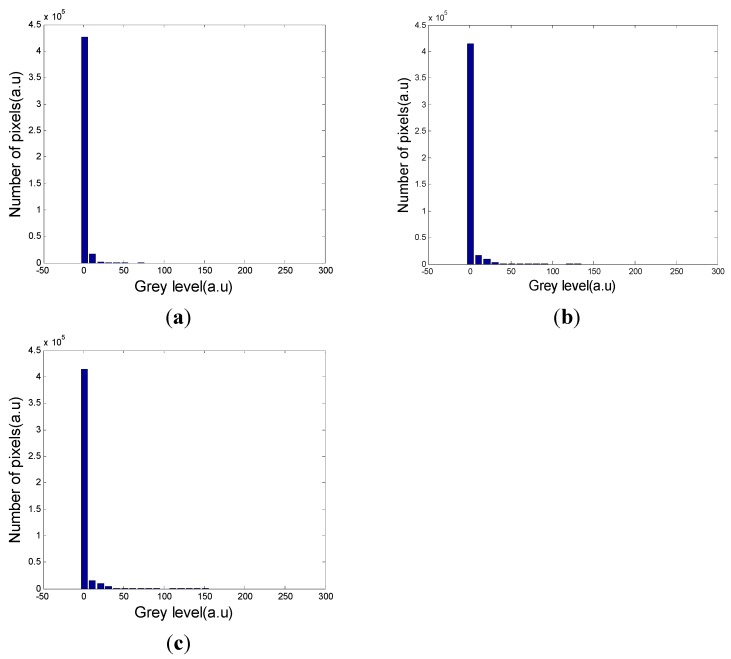
(**a**) Grey level histogram of vehicle passing; (**b**) Grey level histogram of digging. (**c**) Grey level histogram of walking.

Before feature extraction, segmentation is required to separate the event region from the background. The grey level histograms of the three types of signals are shown in Figure 7. As can be seen from the grey level histograms, there is a large difference between the event region and the background, so threshold segmentation is an appropriate method [11].

An artificial threshold will lead to errors, because a high threshold will contain noise and a low threshold will discard information of the characteristics in the region. The Otsu threshold is calculated by the grey level histogram [12]. The main principle of this method is to find the maximum between-class variance threshold. The Otsu threshold regards the event region and the background as two classes. Firstly an initial threshold is set, and classification between the two classes can be calculated using Equation (8). Then a threshold is found which will maximize this formula:

(8) $$\eta =\frac{\sigma_{B}^{2}}{\sigma_{G}^{2}}$$

where $\sigma_{B}^{2}$ is the between-class variance and $\sigma_{G}^{2}$ is the global variance, which is a constant. The best threshold will maximize $\sigma_{B}^{2}$:

(9) $$\sigma_{B}^{2}(t_{best})=\max\sigma_{B}^{2}(t)$$

**Figure 8:**
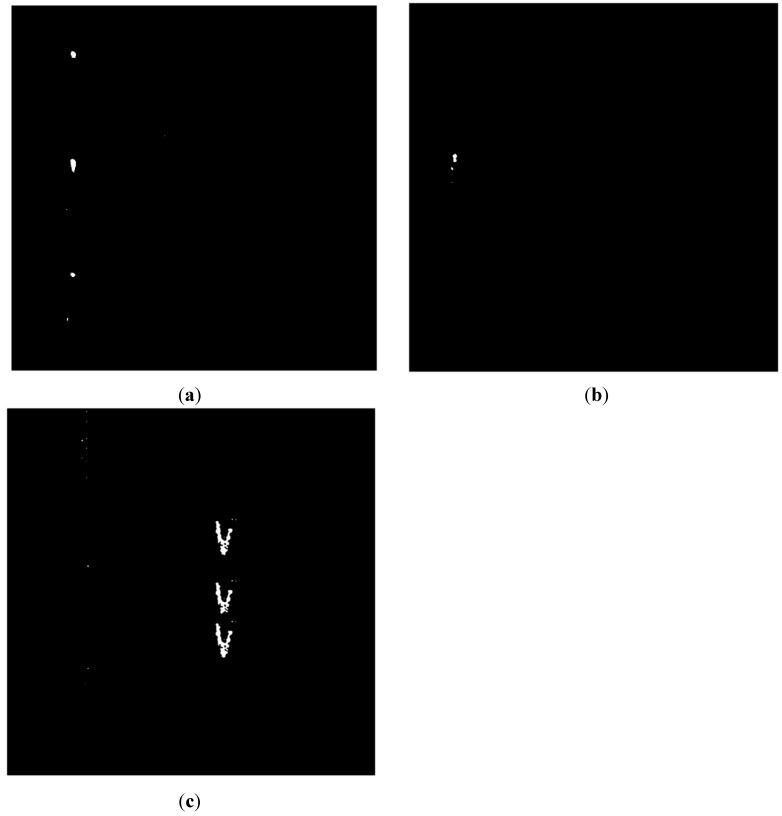
(**a**) Image of walking after threshold segmentation; (**b**) Image of digging after threshold segmentation; (**c**) Image of vehicle passing after threshold segmentation.

$t_{best}$ is the optimal threshold. As can be seen from Figure 8, the segmentation method is effective and satisfactory for our purposes. There are some noise points appearing in the images in Figure 8, which are one order of magnitude smaller than the event region, and are widely dispersed. Median filtering is an effective way to eliminate these noise points [13]. Also, there are some holes in the images that have the potential to seriously impact the effects of feature extraction, so an image dilation strategy was used for filling these holes.

**Figure 9:**
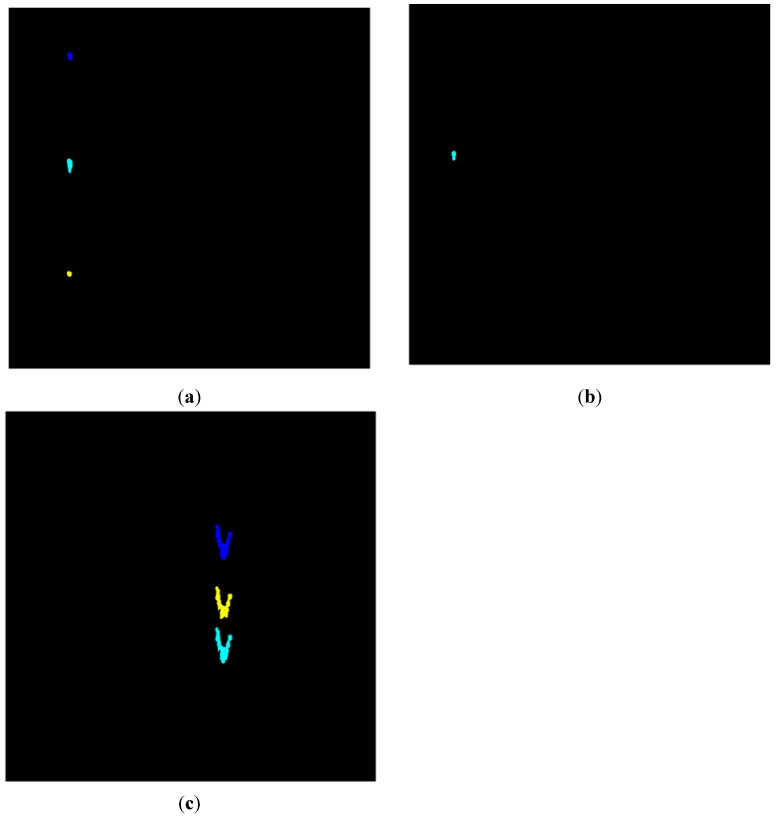
(**a**) Image of walking showing events which are labeled in different colors; (**b**) Image of digging showing events which are labeled in different colors; (**c**) Image of vehicle passing showing events which are labeled in different colors.

Figure 9 shows each event region labeled in different colors. If there is more than one region in an image, the pixels of different regions will be labeled with different values; therefore different colors can be seen within the image.

### 3.2. Feature Extraction

In Figure 9, every marked area is an event region. One region means an event happening once. In our experiment, there are three walking event regions, three vehicle passing regions and one digging event region every two seconds. If the signal is acquired for only one second, there may be only one walking event region, two vehicle passing regions or one digging region. There will be additional event regions within each image when the signal is acquired for a longer time. In order to uniformly process the image, every region in each image is used as a research object instead of the whole image.

**Figure 10:**
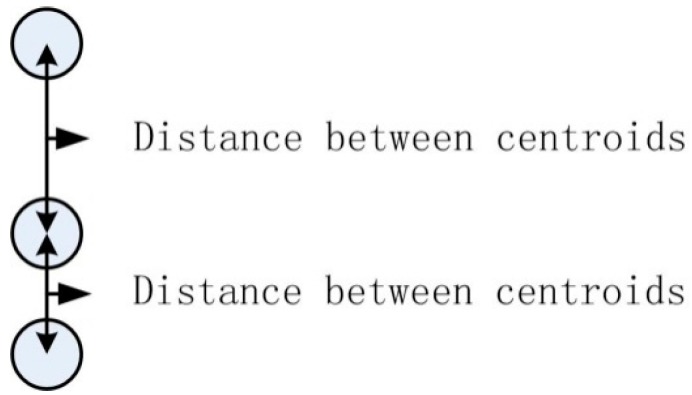
Intervals of centroids.

Firstly, different events have different amplitudes because they exert different forces on the ground, so the amplitude “Amp” of the original signal in a different labeled region is used as a classification feature. Secondly, at the same frequency, the interval of two regions for one event in one image is different from the other events. If one region has two neighboring regions, the shortest interval is chosen as the feature value. The intervals between regions are shown in Figure 10.

The centroid of a different region is first located as $C=[c_{1},c_{2},\cdots ,c_{n}]$, where n is the number of regions in the image. In order to avoid feature extraction errors when multiple events are happening at the same time, the abscissas of the centroids of regions are firstly compared. Two regions are classed as belonging to different events if their centroid abscissas have a sufficient distance from each other. When calculating the intervals between regions, the method will skip a region if its abscissa is far from the previous region. The distance threshold for the abscissa is set to be the minimum distance of abscissas between non-overlapping regions from different events. The distance from the centroid of one region to the centroid of its neighbor in the same event is then calculated and the shortest distance is found using Equation (10):

(10) $$d_{\min1}=\min(\left|c_{i}-c_{i-1}\right|,\left|c_{i}-c_{i+1}\right|)i\in (2,\cdots ,n-1)$$

**Figure 11:**
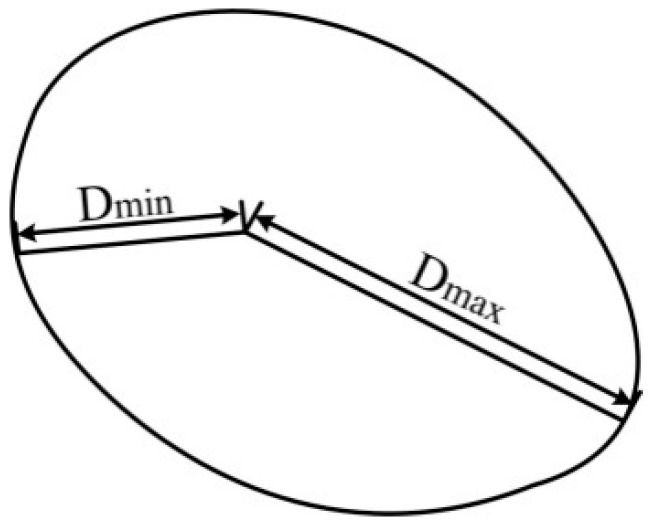
Shape of region.

If there is only one region in an image, the interval feature is set to be a large value. Different events also have different region shapes, for reasons that were described in Section 1. The region where a vehicle is passing is similar to the letter “V”, due to its large energy but short duration. Other events have a lower energy and a relatively long duration time, so many “V” shapes are observed in the region in the time domain and a round shape is formed in the image. The size and roundness of the event regions for walking and digging are both different due to their different energies. The roundness of the region can be used as the shape feature. The process of obtaining the shape of the region is shown in Figure 11. Firstly, the boundary of the region $b_{i}$ must be acquired. In this paper, the boundary can be found using a boundary tracking method [14] which is a simple and efficient method. The distance from the centroid to every point of the boundary is calculated using Equation (11):

(11) $$D_{ik}=\left|b_{ik}-c_{i}|,k\in (1,\cdots ,k\right)$$

In Equation (11), K is the number of boundary points used. The maximum distance *D_i_*_max_ and the minimum distance *D_i_*_min_ can be easily obtained. The shape feature is calculated using Equation (12):

(12) $$S_{i}=\left|D_{i\max}-D_{i\min}\right|$$

A comprehensive description of the region is necessary for recognition; some common region descriptors which can describe differences between events are also used as features [15]. Seven region descriptors are chosen as features in this paper. All features are listed in Table 1.

**Table 1:** Features of an event region.

Feature	Definition
Amp	Amplitude of time-space domain signal
Cen	Minimum interval between regions
Shape	Roundness of the region
Con	Pixel number of the convex hull
Area	Pixel number of the region
Ecc	Eccentricity of ellipse which has the same second moment as the event region
Maj	Length of the long axis of the ellipse which has the same second moment as the event region
Min	Length of the short axis of the ellipse which has the same second moment as the event region
Equ	Diameter of a circle which has the same total area as the event region
Eul	Remaining number of objects, excluding holes in the image region

Due to limiting conditions, a large number of samples of the intrusion event cannot be acquired in a relatively short period of time. Therefore the Φ-OTDR pre-warning system recognition process is a small sample size problem. Fifty samples of each type event were acquired for a limited time from the field environment. One sample represents one event region. There are 150 samples of the three events used for recognition. Ten features of each region mentioned in Table 1 were extracted, and the mean value of each feature is shown in Table 2. There is only one digging region; and the region interval between the other two events is much less than digging. In order to ensure strong separability of features, the centroid interval feature “Cen” of digging is set to 1000. The distance threshold of the centroid abscissas is set by analysis of the region area. In this paper, the distance threshold is set to 25 sample points. If the abscissa distance from the centroid of one region to its neighbor is farther than the distance threshold, the regions are determined to belong to different events. The feature extraction method of centroid interval will therefore skip the region and continue the calculations on the next region.

**Table 2:** Mean value of 10 features of three events.

Event Type	Con	Cen	Area	Amp	Ecc
Walking	279	271.373	245	10.2871	0.7574
Digging	224	1000	205	12.6634	0.8751
Vehicle passing	2413	118.0326	1461	8.5084	0.9118
	**Shape**	**Maj**	**Min**	**Equ**	**Eul**
Walking	8.1041	24.4843	12.9812	17.3192	1
Digging	14.6010	25.9145	10.6407	16.1559	1
Vehicle passing	46.7452	84.0348	40.5031	43.1287	−1

### 3.3. Feature Selection

Comprehensive descriptions can sometimes use too many features, which not only makes the feature extraction method more complex, but also increases correlation between features, resulting in classification errors. A feature selection method to reduce the number of dimensions will increase the recognition speed and accuracy. A scatter matrix calculation method which can evaluate the discrimination of every feature is used in this paper for feature selection. This method will not change the feature attributes and has a very short calculation time. The main concept of the scatter matrix calculation method is to obtain the relationship of the eigenvector distribution in one-dimensional space and select the features with the smallest variance in class and largest distance between classes [16,17]. Firstly, the scatter matrixes of each class and between classes of 10 features of three events are calculated, and then the criterion value of every feature is computed using Equation (13):

(13) $$J=tr\left\{S_{\omega }^{-1}S_{m}\right\}$$

where *S_ω_* is the scatter matrix in the class and *S_m_* is the hybrid scatter matrix calculated using Equation (14):

(14) $$S_{m}=S_{\omega }+S_{b}$$

where *S_b_* is the scatter matrix between classes.

**Figure 12:**
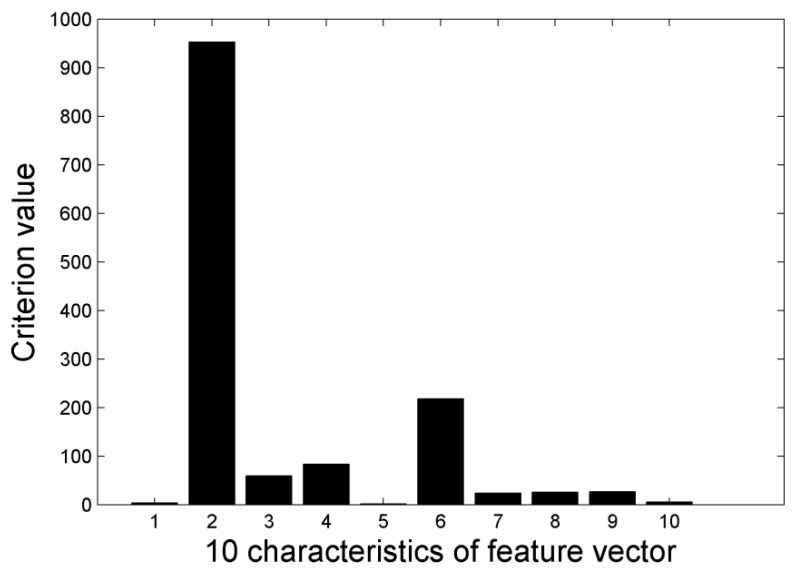
Criterion values of 10 characteristics.

If samples of every class are clustered around the mean value and there is complete separation between classes, the criterion value is highly suitable. The criterion value of every feature is calculated using Equation (13) and features with a larger value are selected as recognition samples. The result of feature selection is shown in Figure 12.

In Figure 12, 10 features are numbered according to Table 2. The first four largest value features are “Cen”, “Shape”, “Amp” and “Area”. The relevance of features is calculated in Equation (15). $x_{nk}$ is the number *k* feature of number *n* sample. n=1,2,⋯,N, k=1,2,⋯,m. In this paper *N* = 150:

(15) $$\rho_{ij}=\frac{\sum \begin{array}{c} N\\ n=1 \end{array}x_{ni}x_{nj}}{\sqrt{\sum \begin{array}{c} N\\ n=1 \end{array}x_{ni}^{2}\sum \begin{array}{c} N\\ n=1 \end{array}x_{nj}^{2}}}$$

First the feature with maximal criterion value “Cen” is selected as $x_{i1}$. Second, the relevance $\rho_{ij}$ between $x_{i1}$ and other *m*−1 features is calculated by Equation (15). Finally, the feature which satisfy the following equation will be selected as $x_{i2}$:

(16) $$i_{2}=\arg\max\{\alpha_{1}J(j)-\alpha_{2}\left|\rho_{i1j}\right|\},\begin{array}{cc} & j\neq i_{1} \end{array}$$

${\text{α}}_{1}$${\text{α}}_{2}$ are the weighting coefficients. In this paper, ${\text{α}}_{1}$${\text{α}}_{2}$ are set to 0.5 respectively. *J* is the criterion value. The other features $x_{ik}$, k=3,⋯,l, can be calculated by Equation (17):

(17) $$i_{k}=\arg\max\{\alpha_{1}J(j)-\frac{\alpha_{2}}{k-1}\sum_{r=1}^{k-1}\left|\rho_{irj}\right|\},\begin{array}{cc} & j\neq i_{r},r=1,2,\cdots k-1 \end{array}$$

The features relevance calculated by Equation (17) is shown in Table 3. $x_{i1}$ is the feature “Cen”.

**Table 3:** Relevance of features.

$x_{ik}$	Feature	Value of $i_{k}$
$x_{i2}$	Shape	159.8173
$x_{i3}$	Area	71.8518
$x_{i4}$	Amp	71.8435
$x_{i5}$	Maj	11.8
$x_{i6}$	Min	11.7677
$x_{i7}$	Equ	11.7333
$x_{i8}$	Eul	7.7
$x_{i9}$	Con	7.6
$x_{i10}$	Ecc	3.6

**Figure 13:**
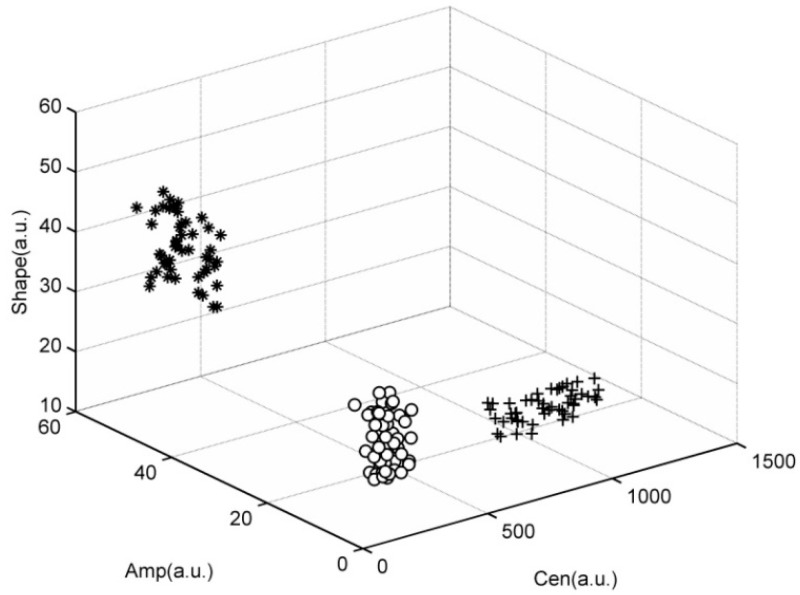
Feature distribution of three events.

It can be seen from Table 3 the minimum relevance four features just are the features which have max scatter matrix value *J*. Therefore the first four features “Cen”, “Shape”, “Area”, “Amp” are finally selected as the feature vector. The features distribution figure of the three intrusion events is shown in Figure 13.

In Figure 13, “o” represents the walking intrusion events, “+” represent the digging events, and “*” represents events due to vehicles passing. As we can see from Figure 13, there is a high dispersity between different intrusion events, and sample points from each type of event are located closely together. Features distribution can verify the separability of features extracted by morphologic method. The effective features can not only improve the recognition accuracy, but also can simplify the design of the classifier.

## 4. Experiments

**Figure 14:**
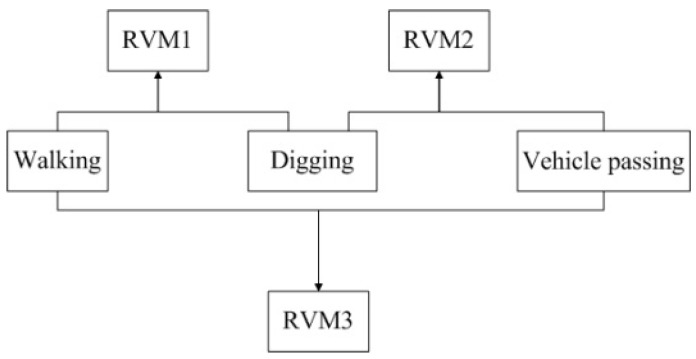
Training process of the three classifiers.

The event samples acquired using the Φ-OTDR pre-warning system are multiclass and the number of samples is small, so the Relevance Vector Machine (RVM) technique is used in this paper. RVM is a machine learning method based on the Bayesian framework. It is sparser than the Support Vector Machine (SVM) technique; hence it has a shorter recognition time and a higher accuracy [18]. This makes it more suitable for use for recognition for an optical fiber pre-warning system [19,20,21]. The Gauss kernel function is used in this paper because of its widely usability and excellent performance. The kernel parameter is usually set between 0 and 1 [22]. Through experimental analysis, it was found that the highest accuracy was obtained when the parameter of the kernel function was set to 0.6. The RVM technique was designed for two-class classification problems; therefore a one-to-one multi-category method is used for recognition of the three events [23]. Each classifier recognizes two classes, so there are three classifiers for recognition of the three intrusion events. Each classifier is trained with two events and the training process is shown in Figure 14.

**Figure 15:**
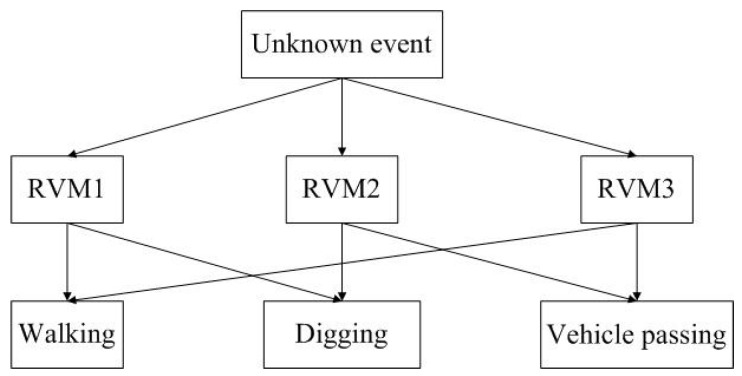
Recognition process of three classifiers.

During the recognition process, an unknown event is recognized by the three classifiers. If two classifiers have the same results, the event will belong to that class. The recognition process is shown in Figure 15.

**Figure 16:**
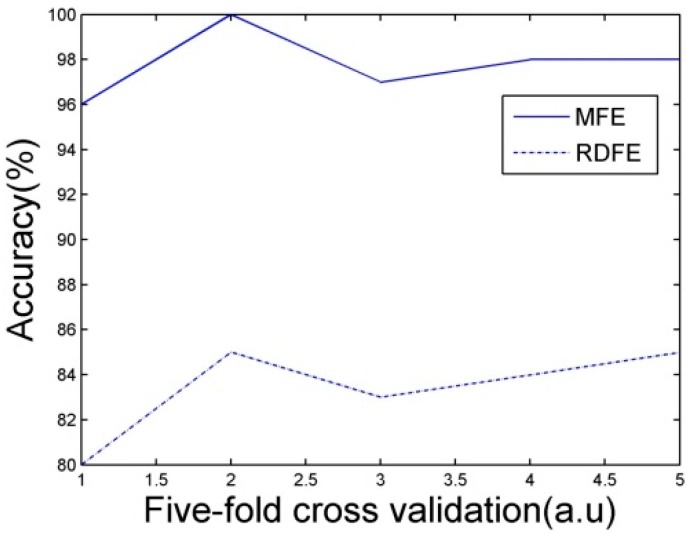
Accuracy of five-fold cross validation.

The performance of the classifiers must be evaluated before recognition. Classifiers are evaluated by a five-fold cross validation. 20 samples from each set of 100 samples of each event are selected for training the classifiers. The accuracy of five-fold cross validation is shown in Figure 16.

In Figure 16, the recognition accuracy of Morphological Feature Extraction (MFE) method with feature selection proposed in this paper is much higher than Region Descriptor Feature Extraction (RDFE) without feature selection. The recognition accuracy can reach over 95%. A single cross validation result is shown in Table 4.

**Table 4:** A single cross validation result.

Sample Model	Walking	Digging	Vehicle Passing
Sample size	20	20	20
Walking	20	1	0
Digging	0	19	0
Vehicle passing	0	0	20
Accuracy (%)	100	95	100

As seen from Table 4, the recognition accuracy of each event also reaches over 95%. This result contains the case where multiple events happen at the same time. There is a recognition error for the digging since the walking signal is mixed with the digging signal due to environmental disturbance. The category of the test sample is known in the test processing, so the classifiers sort the signal into the wrong class. This mistake can be avoided when this method is used in online monitoring, since the class that the signal belongs to is unknown. Traditionally, an event was located in the space domain by a moving average method, and then the event signal was extracted in the time domain. The wavelet method was used for extracting the features [24]. RVM was also used as classifier, and five-fold cross validation was used for evaluating the recognition accuracy. The results of the one dimension signal were shown in Table 4. Identification of the location of the event in the time domain is time-consuming, and since the position accuracy is easily affected by noises, the recognition performance is unsatisfactory. The mean accuracy and recognition time of different methods are shown also in Table 5. When Φ-OTDR is used for safety online monitoring, the algorithm computation time is the recognition time *i.e.*, the time calculated from feature extraction to recognition. When an event occurs, the real-time monitoring system firstly acquires the event signal, then extracts the features proposed in this paper, and finally recognizes the event using the RVM classifiers. The training process was performed before real-time monitoring. In this paper, the recognition time must be within one second.

**Table 5:** Performance comparison of different methods.

Method	Average Precision (%)	Recognition Time (s)
WFE-RVM	80	10.526
RDFE-RVM	85.4	2.169
MFE-RVM	97.8	0.7028

In Table 5, Wavelet Feature Extraction RVM (WFE-RVM) is the traditional recognition method. Region Descriptor Feature Extraction RVM (RDFE-RVM) is the recognition method without feature selection. Morphological Feature Extraction RVM (MFE-RVM) is the recognition method proposed in this paper. Compared with WFE and RDFE, the MFE method proposed in this paper can greatly improve the recognition accuracy. There are only four features after feature selection; therefore the speed of the algorithm also can be increased significantly. The recognition accuracy can reach 97.87%, and the recognition time is within one second, so it meets the requirement of Φ-OTDR online monitoring.

## 5. Conclusions

A time-space signal is regarded as an image in this paper, and a recognition method based on morphological feature extraction is proposed. Image processing technology is used for signal pretreatment, and a scatter matrix is calculated for feature selection. Recognition of pipeline safety events is used to test the performance of the algorithm. The results show that the accuracy and speed are both excellent compared with time domain signal recognition. The proposed method can completely meet the recognition requirements of a Φ-OTDR pre-warning system.
